# Paraptosis Turning Cellular Stress into Therapeutic Weapon for Urological Diseases

**DOI:** 10.34133/research.1156

**Published:** 2026-02-24

**Authors:** Jie Wang, Qi Zhang, Chuanzan Zhou, Zhaojie Lyu, Dechao Feng

**Affiliations:** ^1^ Urology & Nephrology Center, Department of Urology, Zhejiang Provincial People’s Hospital (Affiliated People’s Hospital), Hangzhou Medical College, Hangzhou 310014, Zhejiang, China.; ^2^ Department of Urology, Institute of Urology, West China Hospital, Sichuan University, Chengdu 610041, China.; ^3^ Department of Urology, Institute of Precision Medicine, Peking University Shenzhen Hospital, Shenzhen 518036, China.; ^4^ Division of Surgery & Interventional Science, University College London, London W1W 7TS, UK.

## Abstract

Drug resistance and impaired apoptosis limit durable responses in urological malignancies. Paraptosis is a regulated, nonapoptotic cell death program marked by prominent cytoplasmic vacuolization, typically driven by endoplasmic reticulum (ER) swelling and mitochondrial dysfunction. This perspective synthesizes emerging evidence in prostate, bladder, and renal cancers and proposes a stress-axis framework in which diverse inducers converge into 3 mechanistic classes: (a) proteostasis disruptors that precipitate acute ER stress, (b) ion-handling modulators that trigger lethal mitochondrial Ca^2+^ overload, and (c) redox regulators that amplify reactive-oxygen-species-driven proteotoxic stress. We discuss how natural products, repurposed agents, and delivery platforms can be integrated with chemotherapy, targeted therapy, or immunotherapy to overcome resistance and exploit death-pathway cross talk. Key translational priorities include defining pathway-specific biomarkers, mapping ER–mitochondrion signaling thresholds, and dissecting stress-buffering and cytoprotective autophagy as resistance mechanisms. Clarifying these principles may enable the context-specific exploitation of paraptosis across urological cancers and related diseases, in both primary and therapy-refractory settings.

## Paraptosis in Urological Cancers

Prostate cancer (PCa), bladder cancer, and renal cancer represent 3 of the most prevalent and therapeutically challenging malignancies in the urinary system, characterized by aggressive progression and recalcitrance to standard treatments ranging from androgen deprivation to cisplatin-based chemotherapy and targeted agents [1–3]. As traditional therapies frequently fail to achieve durable responses, paraptosis has emerged as a vital complementary strategy to bypass these resistance barriers [4]. Crucially, current evidence suggests that while the specific triggers for paraptosis vary across these malignancies, they mechanistically converge into 3 distinct classes: (a) proteostasis disruptors, which induce acute endoplasmic reticulum (ER) stress by accumulating misfolded proteins; (b) ion channel modulators, which trigger fatal mitochondrial calcium overload, and (c) redox regulators, which exploit the vulnerability of cancer cells to reactive oxygen species (ROS) accumulation. This integrative classification moves beyond a simple compound-centric view, revealing that paraptosis is not merely a random morphological outcome but a predictable consequence of targeting specific vulnerability nodes within the cancer cell’s stress response network.

**Fig. 1.**
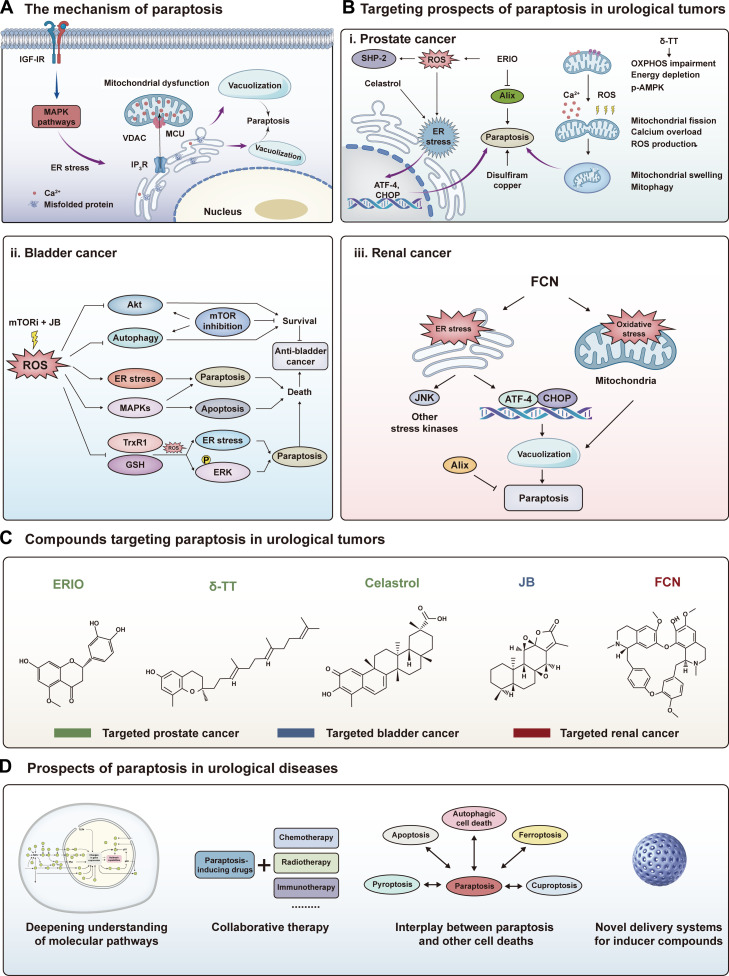
Mechanisms, targeted therapies, and future prospects of paraptosis in urological diseases. (A) The mechanism of paraptosis. Paraptosis is triggered by various cellular stress signals including endoplasmic reticulum (ER) stress, oxidative stress, and mitochondrial dysfunction. These stresses cause vacuolization, culminating in cell death. Key pathways include the mitogen-activated protein kinase (MAPK) pathway, and key proteins involved include the insulin-like growth factor I receptor (IGF-IR), mitochondrial proteins (voltage-dependent anion channel [VDAC] and mitochondrial calcium uniporter [MCU]), and misfolded proteins that contribute to this nonapoptotic form of programmed cell death. (B) Targeting paraptosis in urological cancers. (i) Prostate cancer: Paraptosis can be induced by compounds like celastrol, which activate ER stress and mitochondrial dysfunction, leading to reactive oxygen species (ROS) generation and calcium overload, triggering cell death pathways. (ii) Bladder cancer: The combination of jolkinolide B (JB) with mTOR inhibitors generates ROS, inducing ER stress, paraptosis, and apoptosis via the MAPK and ERK pathways. (iii) Renal cancer: Fangchinoline (FCN) induces ER stress and oxidative stress, activating key stress proteins like activating transcription factor 4 (ATF-4) and C/EBP homologous protein (CHOP) and leading to mitochondrial dysfunction and vacuolization, which drive paraptosis. (C) Compounds targeting paraptosis in urological tumors. Structural representations of bioactive compounds targeting paraptosis, including eriodictyol 5-*O*-methylether (ERIO), δ-tocotrienol (δ-TT), and celastrol (targeting prostate cancer); JB (targeting bladder cancer); and FCN (targeting renal cancer). (D) Prospects of paraptosis in urological diseases. Future research directions for paraptosis therapies including deepening the understanding of the molecular pathways of paraptosis; integration with chemotherapy, radiotherapy, immunotherapy, and new drug delivery systems; and exploration of paraptosis interplay with other forms of cell death (e.g., pyroptosis, apoptosis, and ferroptosis). IP_3_R, inositol 1,4,5-trisphosphate receptor; SHP-2, Src homology region 2 domain-containing phosphatase-2; Alix, ALG-2-interacting protein X; OXPHOS, oxidative phosphorylation; p-AMPK, phosphorylated AMP-activated protein kinase; TrxR1, thioredoxin reductase 1; GSH, glutathione.

In PCa, particularly castration-resistant PCa, the induction of paraptosis offers new hope by primarily targeting the proteostasis network. Natural compounds such as celastrol [5], δ-tocotrienol (δ-TT) [6,7], and eriodictyol derivatives [8], as well as repurposed agents like disulfiram copper complexes [9], consistently demonstrate the ability to trigger paraptosis alongside apoptosis and autophagy, often through ER stress, mitochondrial dysfunction or mitogen-activated protein kinase (MAPK) pathway activation (Fig. 1B (i) and Fig. 1C). Importantly, paraptosis induction has been shown to overcome drug resistance, as in disulfiram copper nanoparticle formulations active against resistant PCa cells, suggesting unique therapeutic advantages [9]. The convergence of oxidative stress, ER dilation, mitochondrial Ca^2+^/ROS imbalance, and stress kinase signaling in mediating paraptosis underlines its mechanistic diversity and potential for broad applicability. Taken together, these findings position paraptosis not merely as an alternative form of cell death but as a complementary strategy that can be co-opted by bioactive compounds to bypass resistance pathways, offering a valuable direction for future PCa therapies.

In bladder cancer, therapeutic strategies often leverage redox regulation to trigger paraptosis. A compelling example is the natural compound jolkinolide B (JB), which has shown potent efficacy. Mechanistically, JB inhibits thioredoxin reductase 1 (TrxR1) and depletes glutathione, leading to excessive ROS accumulation and subsequent ER stress, which drives ERK-dependent paraptotic cell death alongside apoptosis. The pro-paraptotic effect of JB is further reinforced when combined with mTOR inhibitors, which not only potentiate JB-induced ER stress and MAPK signaling but also suppress compensatory Akt feedback activation and cytoprotective autophagy, thereby synergistically amplifying cell death. Moreover, in the context of glutathione peroxidase 4 (GPX4) inhibition, JB overcomes cisplatin and GPX4 inhibitor resistance mediated by TrxR1 overexpression, where dual targeting of GPX4 and TrxR1 augments JB-triggered paraptosis (Fig. 1B (ii) and Fig. 1C) [10–12].

Notably, in renal cell carcinoma, the focus shifts toward multipathway engagement to overcome the metabolic resilience of kidney tumors. In the case of fangchinoline (FCN), its ability to simultaneously activate paraptosis, autophagy, and apoptosis in renal cell carcinoma cells while preserving the defining vacuolization-based morphology of paraptosis independent of caspase activation underscores the therapeutic advantage of engaging multiple programmed cell death pathways. The study demonstrated that FCN-induced paraptosis was tightly linked to redox imbalance and suppression of paraptosis inhibitors such as ALG-2-interacting protein X (Alix), while its complementary activation of autophagic and apoptotic processes ensured robust cytotoxicity. Importantly, the synergistic effects of FCN with paclitaxel not only enhanced programmed cell death but also suggested a route to mitigate drug resistance through pathway redundancy and cross talk (Fig. 1B (iii) and Fig. 1C) [13].

## Research Gaps and Future Directions

Despite the therapeutic promise of paraptosis for urological malignancies, several hurdles still impede clinical translation. The most immediate gap is the absence of pathway-defining biomarkers comparable to cleaved caspase-3 for apoptosis or LC3-II for autophagy [14,15]. In practice, paraptosis is diagnosed largely by morphology, including cytoplasmic vacuolization with ER and mitochondrial swelling, together with the exclusion of other regulated death programs [4]. This morphology-first framework is inherently ambiguous, limits reproducibility across laboratories, and hampers scalable high-throughput screening and pathology annotation. Although regulators such as Alix, voltage-dependent anion channel 1 (VDAC1), and mitochondrial calcium uniporter (MCU) have been implicated [4], the field still lacks a definitive executor or a minimal molecular signature. In addition, many reported “paraptosis-like” phenotypes are induced at supraphysiologic drug concentrations, making it essential to couple biomarker discovery with exposure–response relationships and clinically achievable dosing. Future work should prioritize integrated multi-omics (proteomics, phosphoproteomics, and ubiquitinome profiling) [16] coupled to quantitative imaging to define reproducible paraptosis-enriched signatures. Such signatures could then be adapted into practical assays, such as immunohistochemistry panels, spatial proteomics readouts, and ultimately liquid-biopsy markers to monitor pathway engagement and patient response.

The second gap is mechanistic: the quantitative rules governing organelle cross talk remain poorly defined. Increasing evidence supports a causal role for ER–mitochondrion coupling at mitochondrion-associated ER membranes (MAMs), where excessive Ca^2+^ transfer through the inositol 1,4,5-trisphosphate receptor–VDAC–MCU axis can precipitate osmotic imbalance, mitochondrial depolarization, ROS amplification, and progressive vacuolization [17]. However, the thresholds and kinetics that separate adaptive Ca^2+^ signaling and proteostatic compensation from irreversible death are unknown. Dissecting these thresholds will require time-resolved single-cell measurements of Ca^2+^ flux, membrane potential, and proteostasis capacity, ideally linked to fate mapping. Mechanistic clarity here is not academic: it should expose druggable nodes that selectively amplify lethal ER–mitochondrion flux without broadly poisoning normal tissues.

Third, paraptosis appears uncommon during natural tumor evolution, implying robust buffering and escape mechanisms. Urological cancers frequently upregulate proteostasis defenses, including heat-shock proteins, unfolded protein response effectors, and ubiquitin–proteasome capacity that dampen proteotoxic signals that would otherwise converge on paraptosis [18]. In parallel, cytoprotective autophagy and mitophagy can remove dysfunctional organelles before catastrophic swelling and lysis [19]. These adaptations suggest that effective induction will often require dismantling resistance circuits. Rational combinations should therefore pair paraptosis inducers with inhibitors of stress-buffering pathways (e.g., heat-shock protein blockade, proteasome-adaptive response inhibitors, or autophagy/mitophagy suppression).

From a translational standpoint, the practical value of paraptosis may lie in combination regimens and in apoptosis-refractory disease, where engaging an alternative stress-to-death route could restore therapeutic vulnerability. Natural products with paraptosis-inducing activity (e.g., JB and δ-TT) provide useful starting points, and medicinal chemistry could improve potency, selectivity, and pharmacokinetics. Clinical success will also depend on exposure, stability, and delivery, because many candidates suffer from poor solubility or rapid metabolism. Formulation strategies that increase intratumoral concentration while limiting systemic stress responses will be essential and should be benchmarked against standard-of-care endpoints such as tumor control, renal function, and hematologic toxicity.

A major unresolved question is the immunological consequence of paraptosis. Because paraptosis can culminate in organelle dysfunction and membrane rupture, it may release damage-associated molecular patterns (DAMPs), modulate antigen presentation, and reshape responses to immune checkpoint blockade. Conversely, paraptotic lysis could also promote chronic, tumor-supportive inflammation depending on timing, cell type, and clearance efficiency [20]. Defining DAMP profiles, dendritic-cell priming, myeloid polarization, and T-cell recruitment in immunocompetent urological tumor models is therefore a priority, particularly if paraptosis is to be paired with immunotherapy. These studies should also determine whether paraptosis synergizes with radiotherapy- or chemotherapy-induced immunogenic signals or whether it primarily amplifies immunosuppression via myeloid checkpoints.

Finally, paraptosis-like injury may extend beyond oncology to nonmalignant urological disorders such as acute kidney injury and urinary tract infection, where oxidative stress and vacuolization are frequently observed. Current evidence is largely indirect and confounded by overlapping necrosis and apoptosis, so genetics-informed models and validated biomarkers are required to assign causality. Clarifying when paraptosis is pathogenic versus therapeutically exploitable and developing assays that distinguish these scenarios could broaden the clinical relevance of this stress-to-death program across urological disease contexts. Figure 1D illustrates the prospects of paraptosis in urological diseases.
